# Health–Climate Change–Migration Triad and Elderly Migrant Health in Africa

**DOI:** 10.5334/aogh.5156

**Published:** 2026-07-27

**Authors:** Joseph Etah Oben

**Affiliations:** 1 Research Mentor, Africa Youth for Peace and Development, Free Town, Sierra-Leone

**Keywords:** climate change, migration, elderly health

## Abstract

Climate change is a contributing factor to the high burden of infectious and noncommunicable diseases in Africa, in particular a resource-poor setting already faced with worsening humanitarian and socio-economic crises, as well as a rising incidence and prevalence of noncommunicable and chronic diseases. Thus, examining studies on the triad, climate change–migration–health, would provide more direction on how climate change mitigation can be incorporated into health system planning in Africa. This paper seeks to review studies on the climate change–migration–health triad with a particular focus on those that address elderly health outcomes in relation to the triad and provide guidelines on how governments can enhance their health systems and mitigate climate change to enable the elderly and elderly migrants to cope and age healthily. The author conducted a review of research articles, reviews, and documents using online databases, including Google Scholar, Google, and Web of Science. The following keywords were combined with the Boolean operators AND and OR to conduct the search: ageing population, environmental changes, climate change, gerontology, and the elderly. Limiters were: language: papers published in English on the themes; and period: papers and documents from 1990 to 2025. Documents, papers, and articles were from both regional (African region) and international sources. The author’s search lasted 3 weeks and yielded 44 articles, of which 18 were selected for thematic analysis. Thematic analysis of articles revealed that climate change impacts on the elderly are severe, with the elderly migrants presenting a greater vulnerability; impacts of climate change on African health systems are projected to be severe due to weak health systems. This paper underscores the need for African health systems to integrate data-driven climate change mitigation strategies into health systems and improve community engagement and international collaboration to tackle climate change and health system challenges.

## Introduction

### Background

Climate change effects influence environmental and human health. Environmental changes resulting from the effects of climate change cause ecosystem alterations, a rise in temperature, and in turn affect human health. The rise in temperature resulting in extreme heat, too much sunshine, too much rainfall, floods, and droughts degrades the environment, leading to increased stress on human beings. Human disease conditions, including skin infections, other climate-sensitive communicable diseases, cancers, and noncommunicable diseases, like hypertension and diabetes, have increased in prevalence worldwide due to the rising temperature of the earth as a result of climate change [1, 2].

While all human beings are affected by climate change, the elderly are among those most affected. Scientific evidence suggests that climate change greatly influences healthy ageing and physical and mental functional capacities [3]. It is important to examine the impact of climate change on the environment and the elderly, and how to strengthen and empower the elderly to cope with turbulent climatic conditions [4]. As the elderly constitute one of the most vulnerable groups in the climate change context, elderly migrants are a more vulnerable category of individuals within the climate change phenomenon because of their age, limited movement, and social connections. Elderly migrants, especially those with disabilities, are particularly affected during disasters and humanitarian crises [5]. Examining existing global health policies on elderly well-being and resilience in the face of climate change problems is also a relevant approach to addressing the impact of climate change on the elderly to develop more actionable and sustainable policy guidelines [6, 7].

## Problem Statement

Global health policy frameworks have often focused on the prevention of infectious disease transmission across borders. Environmental influences on human health have often been overlooked. Not until recently that climate change influenced a new twist on cross-border disease control strategies. Yet, the health of vulnerable elderly migrant populations on the African continent in the context of the climate change phenomenon has not been taken into account, or very little attention is paid to the elderly migrant health conditions in the face of climate change [8, 9]. Nevertheless, the increasingly alarming effects of climate change on human health, especially on the elderly, automatically compel the integration of strategies to mainstream ageing and elderly healthcare needs in health system planning to strengthen elderly people’s ability to live and age healthily. The World Health Organization (WHO) [9] seventh report in the Global Evidence Review on Health and Migration (GEHM) series highlights the need for migrant inclusive and climate-resilient health systems [10]. According to the WHO [1, 10], climate change effects are profound on the elderly migrants than any other age group and challenge global health systems to re-strategize and re-orient to contain these demanding sets of requirements, as climate change effects and migrant health are interlinked, exacerbating the migration, climate change, and health issues on migrant-affected populations.

Anu et al. [11] reported the direct and indirect impact of climate change on the elderly, revealing that over 80% of African elderly people in Zambia live below the national poverty line and are disproportionately impacted in a warming climate. Direct impacts of climate change result from severe climate change events such as extreme weather conditions, air pollution, and food insecurity and quality. These are severe for the elderly, especially elderly migrants. This indicates that the health of elderly people in Africa, and by implication the health of elderly migrants, is not a priority in African health systems, as the WHO [10] highlighted: Health systems that prioritize and empower the ageing and elderly tend to be resilient in the context of climate change, support healthy ageing, and produce healthy elderly people.

This literature underscores the need for global health systems to integrate elderly care needs across borders while considering their resilience within the climate change phenomenon impacting the lives of elderly migrants gravely. That is, global health systems have to extend beyond cross-border measures of preventing communicable disease spread to integrate actions that would cause elderly migrants to cope and age healthily within this era in which the climate change phenomenon–migration–health triad is manifesting [12].

## Findings and Recommendations

### Findings

A growing body of scientific evidence indicates that the impacts of climate change on the elderly, especially elderly migrants, are graver than on any other age group. Consequently, this challenges global health diplomacy’s long-term border-focused strategies of communicable disease prevention to extend beyond and embrace and tackle the impact of climate change on the elderly through climate change mitigation, as global health diplomacy and climate change influence affect elderly migrants compared with any other age group [1, 10].

The major findings documented in works reviewed on this triad of global health–climate change–migration are outlined in Table 1.

**Table 1:** Summary of findings within the climate change–migration–health triad.

AUTHOR(S)	TITLE	FINDING WITHIN THE CLIMATE CHANGE–MIGRATION–HEALTH TRIAD
UNFCCC [7]	United Nations Framework Convention on Climate Change	The Convention highlighted the need to mitigate climate change to prevent its future profound impacts on humans to foster coping and development, including food production and economic development in a natural and sustainable manner through the reduction of greenhouse gas.
Carnes et al. [13]	Impact of climate change on elder health	They highlighted that as human life expectancy increases, so does the number of elderly people. They also noted that the elderly, in particular, will experience severe health consequences in cases of serious environmental stress due to climate change.
Kalaiyarasi and Rajkumar [14]	Impact of climate change on elderly health	They reported that climate change is a major public health threat resulting from the effects of extreme weather events such as floods and drought, rising sea levels, and increasing temperatures. They highlighted that health systems have to incorporate climate-resilient strategies to support the elderly as their health and well-being can be gravely affected in the climate change context.
IPCC [6]	Summary for all climate change 2021	They underscored the fact that human activities drive climate change, which causes severe impacts on humans and the environment. They noted that increasing greenhouse gases, rising global temperatures, and rising ocean levels are some of the environmental impacts of climate change. They revealed that humans face severe environmental and health challenges in coping with and mitigating climate change. They recommended urgent and sustained approaches to mitigate the effects of climate change today to prevent future catastrophes on humans and the environment.
Montoro-Ramírez et al. [15]	Effects of climate change in the elderly’s health: a scoping review protocol	They underscored the fact that climate change is a global issue that affects human health, particularly vulnerable groups like older adult migrants. They proposed data-driven approaches for meeting the care needs of the population in the context of climate change [15].
National Oceanic and Atmospheric Administration [16]	National Oceanic and Atmospheric Administration	They reported that people and other living things in the environment around the world are affected by the manifestation of climate change today. The document suggested that communities should invest in resilient infrastructure that can resist future climate risks.
Anu et al. [11]	Effect of Climate Change on Health in Older Persons	They noted the increasingly population of the elderly (over 65) and highlighted that the elderly are greatly affected by climate change, with worsening disease conditions when they are exposed to climate-change-related stresses.
World Health Organization [1]	Climate change	The World Health Organization (2023) reported that climate change is directly affecting humanitarian crises and noted that 3.6 billion people are already dwelling in climate-change-prone zones. They also highlighted that between 2030 and 2050, it is anticipated that climate change will cause an estimated number of 250,000 deaths per year as a result of malaria-related diseases, heat stress, diarrhea, and malnutrition, indicating that the consequences of climate change will be grave in developing countries with weak health infrastructure, as they will be unprepared to cope and respond. The WHO suggested climate-resilient and migrant-inclusive health systems.
Matlakala et al. [5]	Vulnerability of elderly people during climate-induced disasters in Sub-Saharan Africa: a scoping review	The scholars reiterated immediate action regarding specific research and development of community-engaged actions on the enhancement of coping mechanisms and well-being of older people, especially those with disability experiencing climate-influenced disasters. They noted a lack of studies examining the interplay between climate change and elderly health.
Hosseini et al. [17]	Climate crisis risks to elderly health: strategies for effective promotion and response	The scholars reported that climate change affects the health and well-being of older people directly and indirectly. They recommended tailoring risk response strategies, and also awareness and standardization to improve risk management for the well-being and resilience of older people in the climate change context.
Prina et al. [3]	Climate change and healthy ageing: An assessment of the impact of climate hazards on older people	They reported that there is an urgent need to address the effects of climate change on the health of the general population and re-examine the impact of climate change on the health of elderly people. They recommended that it is important to identify ways to mitigate and adapt to climate change, engaging older people since it affects their health and healthy ageing.
Santino et al. [9]	Climate change and health: displaced and migrant populations must be included	They recalled that the link between climate change and health was recognized in 2023. The scientists explained that migration and displacement are naturally linked to health and climate change. However, they highlighted that little is known of the climate–migration–health triad. Furthermore, they noted an enormous scale of migration and displacement resulting from climate change, revealing that 24 million people are displaced by natural disasters annually, and this number is projected to increase to 1.2 billion people by 2050.
Foreignpolicy [18]	Strengthening global health in the face of climate change	They revealed that climate change is posing challenges on health and well-being. They suggest that health systems need to scale up action against climate change in a sustainable way to adapt and mitigate climate change, especially for low- and middle-income countries.
Haciibrahimoglu [8]	An Analysis of Climate Change: Identifying Research Trends in Global Warming	The researcher reported that global warming and climate change are pressing issues of the present era. They underscored the ecosystem effects of climate change with unavoidable loss of valuable species and noted that, on society, effects could lead to displacement or migration and affect human health. The researcher called for urgent collaborative action to adapt to climate change and global warming and mitigate climate change.
Lancet Countdown [2]	2025 Report—Lancet Countdown	The report of 128 experts draws attention to the worsening health effects of climate change, with millions of preventable deaths yearly resulting from increasing greenhouse gas emissions, failure to adequately adapt to climate change, and fossil fuel dependence. They noted that environmental conditions and planetary systems are increasingly being destabilized by climate change, worsening human health outcomes.
Morrison and Bliss [4]	The health-climate-security triad	The researchers noted that the climate change phenomenon is increasingly being recognized as a health crisis, resulting in increased pathogen threats, morbidity and mortality rates. They emphasized the importance of cooperation and the need for data-driven strengthening of climate-resilient health systems.
Stone [12]	Beyond borders: Collaborative approaches to global health security	They highlighted that in the midst of other issues, climate change poses significant threats to human health and stability. They noted that health systems across the world are weakened by security threats and inequality in resource allocation. They proposed that the world can establish global health security through lessons learnt from challenges and successes of past collaborative approaches to achieve resilient health systems against emerging health threats such as climate change.
World Health Organization [10]	Climate change and global health: WHO calls for migrant-inclusive and climate-resilient health systems	They reported that climate change is a risk factor for human displacement, as it worsens the situations of vulnerable populations, including women, children, and the elderly, resulting in enormous stress on health systems. The WHO suggested migrant inclusive and climate-resilient health systems.

**Summary on research trends on climate change–migration–health triad by year**

- Table 1 shows a steady rise in the number of studies on climate change, migration, and health, though beginning sporadically from 1992 to 2025, indicating that the subject is increasingly gaining attention within the climate change–health–migration scientific community and related organizations.
- Nevertheless, this rising trend from the 1990s to 2025 is uneven, as there are gaps between the early 1990s and early 2010s, contributing only 11.1% of the total research output, with approximately 20 years in a row having no studies on the subject. By contrast, 2024 and 2025 combined contributed 44.4% of the total research output. Meanwhile, 2022 and 2023 contributed 16.7% and 11.1%, respectively, and 2020 contributed only 5.6%.
- The surge in 2024 and 2025 indicates increased research activity within the domain that involved international organizations, individual researchers, institutions, and research groups working collaboratively, while the drop in the gap years highlights limited research activity requiring urgent action to uphold research activity in the climate change–migration–health domain to contribute in strengthening health systems and enhancing health outcomes, particularly among the elderly and elderly migrants.

### Highlights of the findings

- **Increased morbidity and mortality among the elderly population:** The reviews have revealed that the elderly are most affected by environmental changes, resulting from climate change. They highlight that elderly people do not easily adapt to these changes, and as a consequence, most elderly people die from extreme weather conditions, including too much rainfall, too much sunshine, increased air pollution, and hunger and starvation resulting from poor food quality and safety.
- **Increased vulnerability among the elderly, especially the elderly migrants:** As climate change consequences affect the elderly gravely, and as their health needs are not integrated in the health systems, they become very vulnerable and succumb to the effects.
- **Weak health systems in low- and middle-income countries:** Health systems in low- and middle-income countries are weak and unprepared to cope with future climate change impacts, resulting in poor health outcomes for elderly people, particularly elderly migrants.
- **Climate change is a huge public health threat:** Climate change, health, and migration are inextricably linked, and the interplay between climate change and migration has a huge impact on the health of migrants, especially elderly migrant. Moreover, climate change is an important contributor to humanitarian crises, resulting in human displacement while leaving others in climate-vulnerable circumstances. The World Health Organization [1] noted that climate change is directly affecting humanitarian crises and reported that 3.6 billion people are already dwelling in climate-change-prone zones. Similarly, Santino et al. [9] reported that the link between climate change and health was approved in 2023, and revealed that 24 million people are displaced by natural disasters annually, and this number is projected to increase to 1.2 billion people by 2025, especially in developing countries.

## Recommendations

The following recommendations would contribute in reducing the severe consequences of climate change:

- Engaging community members and the elderly in climate action and increasing the awareness of the elderly and other community members on climate change should constitute part of adaptation and mitigation strategies.
- Building a climate-resilient and migrant-inclusive health infrastructure should consider the prediction, mitigation, adaptation, and the engagement of the elderly in the fight against climate change. It is important to tailor climate change mitigation actions to meet the needs of the population, especially the elderly. For example, the standardization of risk response approaches for the older population.
- Global health diplomacy should go across borders to foster cross-border collaboration to enhance health system resilience, humanitarian crisis prevention, and emergency response to provide opportunities for the elderly, particularly elderly migrants, to cope in the context of climate change and age healthily.
- National, regional, and international health systems should respond to climate change challenges through data-driven approaches. Using lessons learnt from the successes and failures or challenges of past collaborations would inform on the way forward.

## Implications and Conclusions

The increasingly growing consequences of the climate change phenomenon on humans include more loss of lives and property, ecosystem alterations, and loss of certain rare and important plant and animal species. This also highlights that humans, the elderly (including elderly migrants), cannot adapt to these changes faster, so the death toll among the elderly will be higher, and the ageing population will not age in good health and dignity. This implies declining life expectancy, stifling progress toward the attainment of Sustainable Development Goal (SDG) 3: Good Health and Well-Being. While life expectancy is increasing globally, it presents a greater challenge for health systems to cater to the needs of the elderly and other vulnerable groups, including elderly migrants.

Therefore, this is a call to action regarding the potential negative effects that the interplay of migration, climate, and health can have on the third generation. Simply stated, as the elderly constitute the most vulnerable group in the face of climate change, the climate–migration–health interplay impacts the ageing and the elderly the most.

While enormous efforts are being made to address climate change causes and mitigate its effects, more government-subsidized activism, including migrant-inclusive health systems and cross-border cooperation among global health systems, is dire. This will not only help mitigate the negative impact of the climate change–migration–health triad, but will also underscore and address the major issues plaguing the ageing and elderly, particularly elderly migrants, in this climate change context. As a consequence, this will result in a globally sustained approach to health system resilience within the climate change–migration–health triad that is sustainable and reliable for African governments and beyond.

## Data Availability

Data sharing is not available for this study as data were neither generated nor analyzed.
